# Knowledge-Based Attitudes of Medical Students in Antibiotic Therapy and Antibiotic Resistance. A Cross-Sectional Study

**DOI:** 10.3390/ijerph18083930

**Published:** 2021-04-08

**Authors:** Tomasz Sobierajski, Beata Mazińska, Monika Wanke-Rytt, Waleria Hryniewicz

**Affiliations:** 1 Faculty of Applied Social Sciences and Resocialization, University of Warsaw, Krakowskie Przedmieście 26/28, 00-927 Warsaw, Poland; 2 Department of Epidemiology and Clinical Microbiology, National Medicines Institute, Chełmska 30/34, 00-725 Warsaw, Poland; b.mazinska@nil.gov.pl (B.M.); w.hryniewicz@nil.gov.pl (W.H.); 3 Department of Pediatrics with Clinical Assessment Unit, Medical University of Warsaw, Żwirki i Wigury 61, 02-091 Warsaw, Poland; mowanke@gmail.com

**Keywords:** antibiotic, antibiotic resistance, antibiotic misuse, education, knowledge, medical students

## Abstract

We aimed to evaluate the knowledge-based attitudes of antibiotics and antibiotic resistance among medical students of Medical University of Warsaw using the questionnaire prepared by the study’s authors. In May–June 2018, we carried out a cross-sectional study among the students of all years, embracing 291 respondents. The students were divided into two groups: A (students in their first to third years) and B (students in their fourth to sixth years). Our study has shown that students are aware of the dangers of antibiotic resistance, seeing the leading cause as antibiotic misuse. We have shown that they are also aware of their insufficient knowledge and believe that more antibiotic therapy classes should be included in the curriculum of Medical University of Warsaw. Our questionnaire also focused on attitudes towards antibiotics based on knowledge. One in four respondents (23.7%), based on their knowledge, negated antibiotic therapy ordered by a doctor in the event of their illness, and four in ten (40.9%) in the occurrence of disease of a family member or friend. The vast majority of students (92.4%) would like to broaden their knowledge on antibiotic therapy. However, only one-fifth of students have heard about the European Antibiotic Awareness Day campaign. We recommend increasing the number of hours on antibiotic therapy and resistance education combined with topics on hand hygiene.

## 1. Introduction

Rapidly growing antibiotic resistance among bacterial pathogens is one of the most important medical global concerns limiting effective treatment and leading to increased morbidity and mortality [1,2]. This is particularly worrisome since the supply of new effective antimicrobial drugs does not fulfil present needs. Resistance has been detected in all major pathogens and is not limited to nosocomial settings but also concerns community-acquired infections [3].

Several studies indicate that the most critical factors driving resistance include excessive antibiotic consumption and poor hygiene [4,5].

WHO and several international and national public health organizations issued a set of recommended actions to be taken to limit the emergence and further spread of resistance [6,7]. A so-called “One Health” approach was strongly advised by the EU and WHO, which requires combined actions to rationalize antimicrobial use both in humans and in animals [7]. The actions should include education of medical and veterinary professionals, the general public, with particular emphasis on farmers, and politicians. 

Additionally, continuous monitoring of resistance in major pathogens and antibiotic consumption should be strengthened, and the data obtained broadly disseminated. Regular access to modern diagnostic tools should be facilitated, and investment into new antimicrobial therapy and vaccines should be encouraged. To regularly bring antimicrobial resistance to the attention of a broad public, 12 years ago, the EU established European Antibiotic Awareness Day (EAAD) on 18 November. This initiative was also later taken by WHO, which established World Antibiotic Awareness Week (WAAW), which takes place every November. It promotes global education on antibiotics, how they should be used, and warns on the growing risks of antibiotic resistance and its consequences.

As well as dentists, physicians play an essential role in limiting antimicrobial resistance due to more rational prescribing. In our previous study, we have already shown some gaps in the knowledge on antibiotics of Polish dentistry students before graduation [8]. They are a group of professionals who also contribute to the overuse of antibiotics, and the questions addressed to them in our study concerned the principles of antibiotic therapy and the awareness of antibiotic resistance [8]. The present study refers to medical students at the different years of studies and their knowledge-based attitudes towards antibiotics and the problem of antibiotic resistance. There are a large number of publications on this topic from various countries, and we refer to them in the discussion section of the paper. It was demonstrated in some studies that up to 70% of antibiotics are unnecessarily prescribed, which, with combined efforts, can be significantly decreased [9,10]. Many international studies have shown that the most efficacious way to improve antibiotic prescription involves direct education including communication training, access to therapeutic recommendations, antibiotic stewardship programs, access to point-of-care tests (POCT), and more time for patient evaluation at the physician’s office [11,12,13]. 

Poland is one of the leaders among EU countries in antibiotic consumption in the community and the country with one of the highest resistance rates among major bacterial species. In 2019, the average consumption of antibacterials for systematic use (ATC group J01—subgroup of the Anatomical Therapeutic Chemical Classification System) per 1000 inhabitants per day in the community in Poland was 22.2 DDD (defined daily dose) compared with EU/EEA 18.0 DDD (country range: 8.7–32.4) [14].

This study aimed to evaluate medical students’ knowledge at various medical education levels concerning antibiotics and resistance and determine gaps in their education. Medical students, as future physicians, will be prescribing antibiotics in their everyday practice. That is why it is so important to know their knowledge on antibiotics, their approach to their use, as well as their methods of dealing with the challenges of antibiotic resistance.

## 2. Materials and Methods

### 2.1. Study Design and Population

The survey was conducted in May–June 2018 among medical students of the Medical University of Warsaw. The students were divided into two groups: A, 1–3 years and B, 4–6 years. The division was due to the fact that students in years 1–3 have only preclinical classes and students in years 4–6 have postclinical classes, and pharmacology classes and the course on antibiotic therapy and the phenomenon of antibiotic resistance takes place in the 4th year of study. 

The survey was an auditory survey, which involves gathering a selected group of people at a specific location and giving them a questionnaire to fill in. The questionnaire was completed by students during lectures and after exams. Participation in the study was anonymous and voluntary. Then, the data from the sheets were manually entered into the statistical program.

### 2.2. The Questionnaire

The research was carried out using the questionnaire, which was prepared by the study’s authors. 

The structured questionnaire consisted of 26 items in 5 parts: (a) socio-demographic characteristics of the respondents (2 items); (b) perceptions and attitudes about antimicrobial use (9 items); (c) antimicrobial education and educational needs on antibiotics (5 items); (d) source of information about antibiotics (1 item); attitudes towards antimicrobial (3 items); (e) knowledge about antimicrobial resistance (AMR) (6 items). The translated questionnaire has been included in supportive information (Supplementary Materials File S1).

The questionnaire used in this study was an original questionnaire created especially for this study, based on sociological methodology and knowledge. The questionnaire including closed questions, multiple-choice questions, and questions using variations of a 6-point Likert scale, where individual points of the scale, depending on the wording of the question, meant one end of the scale: 1—definitely no/strongly disagree/decisively insignificant degree/decisively insignificant impact, and the other end of the scale meant 6—definitely yes/strongly agree/decisively significant degree/decisively significant impact). In assessing antibiotics knowledge, we used a 6-degree scale, where the lowest degree on scale 1 meant “very bad”, and the highest degree on scale 6 meant “very good”. Question 15: “Would you like to broaden your knowledge about antibiotics” and question 19: “Do you think that the issue of antibiotic resistance of microorganisms is a significant problem” were filter questions.

In order to check the form, clarity, and validity of the questions in the questionnaire, we conducted a pilot survey on a selected group of students and residents of Medical University of Warsaw. 

### 2.3. Statistical Analysis

The survey results were analyzed statistically using IBM SPSS Statistics for Windows 19.0 software package. Data collected were expressed as frequencies and percentages. To test normality, the Kolmogorov–Smirnov test was used. To identify the differences between the study groups, Pearson’s Chi-squared tests, Fisher test, and Mann–Whitney test were used for not normal variables. Likert scale question responses were tested using the Mann–Whitney test. We used a two-tailed test significance level of 0.05. 

Adjusted odds ratios (ORs) with corresponding 95% confidence intervals (CIs) were calculated. In the study analyses, we adjusted for gender (controlling variable). For all tests, *p*-values of 0.05 or less were considered to be statistically significant.

### 2.4. Ethical Considerations

The participants were informed and assured of anonymity and confidentiality. Based on the data collected, analyzed statistically, and presented below, it is impossible to identify the survey participants.

The Ethics Committee approved the study of Medical University of Warsaw (registration number: AKBE/108/2018).

## 3. Results

### 3.1. Study Participants

Out of 968 eligible medical students, 291 responded to the survey, 70% women and 30% men. The sample group was ethnically homogeneous. The response rate was 30%. Group A (1–3 years of study) = 158, group B (4–6 years of study) = 133. 

### 3.2. Perceptions and Attitudes about Antimicrobial Use

Forty-six percent of students (*n* = 133) had used antibiotics within the previous 12 months, 20.1% (*n* = 58) used antibiotics over a year ago but no more than 2 years ago, and 33.9% (*n* = 98) used antibiotics more than 2 years ago. 

The vast majority of respondents (89%) stated that they obtained their last course of antibiotics from a healthcare professional (Table 1): 50 % from a family practitioner, 31.1% from a physician of other specialties, and 7.7% from a dentist. Four percent (4.2%) had acquired the antibiotics from friends or family; 3.8% used leftovers from previous therapy. Students of the older age group more often used antibiotics obtained from friends or a family member (7.6% vs. 1.3%, *p =* 0.008, OR = 6.315, 95% CI = 1.354–29.461). 

The prevailing reasons for taking an antibiotic were pharyngitis (24.9%), bronchitis (12.8%), sore throat (12.1%), cough (8.3%), sinusitis (8.3%), otitis (8.3%), pneumonia (7.6%), urinary tract infection (7.6%), and common cold (6.6%) (Table 1). 

There were significant differences between student groups regarding the reasons for taking antibiotics for sore throat and diarrhea. The students in group A were more likely to use antibiotics for a sore throat than students in group B (17.2% vs. 6.1%, *p =* 0.004, OR = 0.310, 95% CI = 0.136–0.709). The vast majority of students used the full dose of an antibiotic prescribed (85.8%). Relief of symptoms (26.8%) and being advised by a physician/dentist were the most common reasons to stop antibiotic therapy (24.4%). 

The vast majority of students in group A indicated antibiotics as a first-line treatment in acute bronchitis (66.5% vs 19.5%, *p <* 0.001, OR = 0.123, 95% CI = 0.071–0.211). Group B students more often considered antibiotic as a first-line treatment in urinary tract infections (82.7% vs. 69%, *p =* 0.010, OR = 2.252, 95% CI = 1.269–3.999) and borreliosis (85.7% vs. 70.9%, *p =* 0.002, OR = 2.463, 95% CI = 1.355–4.475). The differences were not statistically significant between group A and group B for responses concerning pneumonia (86.7% vs. 88.7%, *p =* 0.603), acute otitis media (64.6% vs. 57.9%, *p =* 0.306), sinusitis (40.5% vs. 33.1%, *p =* 0.192), and sore throat (24.7% vs. 15.8%, *p =* 0.062).

### 3.3. Sources of Information

The main sources of knowledge of antibiotics mentioned by all students included professional medical literature 48.5%, Summary Product Characteristics (SmPC) 46.7%, physicians 37.8%, and Internet 23.8%. 

For every second examined person (48.5%), professional literature is a significant source of antibiotics knowledge. This source is used by 51.1% of B group students and 46.2% of A group of students. There is a link between the year of study and the source of information on the use of antibiotics by students: at the doctors (*p =* 0.024), on the Internet (*p =* 0.016), and in the drug leaflet (*p =* 0.010). SmPC is an important source of information about antibiotics for 46.7% of the respondents. The information contained therein is more significant for group A than for group B students, 52.5% and 39.8%, respectively. For every third examined person in total (37.8%), a physician is a significant source of information about antibiotics. This source is more often indicated by group A students than by group B, 44.3% and 30.1%, respectively.

Information on the Internet is a significant source of information on antibiotics for every fourth examined person in total (23.8%). This source is significantly more frequently relied upon by group B students than by group A students, 28.6% and 19.7%, respectively.

### 3.4. Education

Students were also asked whether they were taught about the growing problem of antibiotic resistance during their studies: 86.7% of group A students and 99.2% of group B students gave a positive answer (*p <* 0.001) (Table 2). Students of older age groups were more likely to believe that their medical studies contributed to their broader knowledge of the use of antibiotics (*p =* 0.001). 

There is a relationship between the year of study and students’ knowledge of the lack of effectiveness of antibiotics against viruses (*p =* 0.017), including colds (*p =* 0.001) and the flu (*p =* 0.005). Regarding the efficacy of antibiotics against viruses, 91.1% of group A students marked answers that they strongly disagree with this statement, as did 97.7% of group B students. Older-year students more often strongly disagreed that antibiotics are effective against the common cold (78.9% vs. 62.7%). However, it should be noted that 4.4% of group A and 3.1% of group B students agreed that antibiotics are effective against the common cold. Regarding the question about the efficacy of antibiotics against the influenza virus: 3.1% of group B students and 6.3% of group A students indicated that they agree with the statement that antibiotics are effective against influenza.

The vast majority of students strongly agreed that the incorrect use of antibiotics can make microorganisms resistant: for group A students, it was 90.5%, and for group B students, it was 98.5%. 

No statistical significance was obtained in whether the use of antibiotics will make a person resistant to them; however, as many as 17.3% of A and 15.1% of B students agreed with this statement. 

### 3.5. Attitudes Based on Knowledge

Eight out of ten respondents positively assessed their knowledge of antibiotics (81.1%). The students of group B evaluated their knowledge of antibiotics slightly better than group A, 84.2% and 78.4%, respectively. However, 6.5% of the respondents assessed their knowledge of antibiotics as “very good” (the highest on the scale). On average, one in five students (18.9%) assessed their knowledge of antibiotics negatively: 21.5% in group A and 15.8% in group B. 

There is a correlation between the knowledge acquired during medical studies by students of years A and B and the negation of the therapy prescribed by a doctor in the case of own disease (*p =* 0.002) and illness of a family member/friend ((*p* < 0.001) (Table 2).

One in four respondents (23.7%), based on this knowledge, negated the antibiotic therapy ordered by a doctor in case of their illness, and four in ten (40.9%) in case of disease of someone from their family or friends. Group B students negated the antibiotic therapy prescribed by another doctor in case of their illness twice as often as group A, 32.3% and 16.5%, respectively. In the case of a disease of a close relative from family or friends, the antibiotic therapy recommended by a doctor was also more often negated by group B students than by group A, by 53.4% and 30.4%, respectively. 

Three out of ten students (30%) would start the recommended antibiotic treatment fully trusting the doctor who prescribed it. Two thirds (65.5%) of the respondents would check the antibiotic and recommendations in another source before starting the treatment, not fully trusting the doctor who prescribed the antibiotic. Information about recommended antibiotic therapy in another source would be sought more often by students of group B than by students of group A, by 70.5% and 61.4%, respectively. A total lack of trust in the antibiotic therapy prescribed by the doctor and relying only on their knowledge concerned 4.5% of the total number of respondents.

From the group of students who would like to broaden their knowledge about antibiotics’ use, the most frequently indicated topic was the general principles of rational use of antibiotics—80.3% of the total number of respondents. Other issues that students would like to explore (according to the level of indications) were as follows: information on the causes of antibiotic therapy failure (60.2% of the total indications; 61% for group A and 59.3% for group B); information on the antibiotic resistance of respiratory tract infections (58% of total indications; 57.5% for group A and 58.5% for group B); information on the antibiotic resistance of urinary tract infections (45.4% of total indications; 49.3% for group A and 40.7% for group B); information on the antibiotic resistance of gastrointestinal infections (39.8% of total indications; 43.8% for group A and 35% for group B); information on the diagnostic potential of microbiological infections (38.3% of total indications; 44.5% for group A and 30.9% for group B, *p* = 0.022); information on the antibiotic resistance of skin and soft tissue infections (39.8% of total indications; 46.6% for group A and 31.7% for group B, *p* = 0.013); information on bacterial infections of the mouth and oral cavity (28.4% of overall indications; 30.1% for group A and 26.2% for group B).

### 3.6. Knowledge about AMR (Antimicrobial Resistance)

According to the respondents, three elements have the most significant influence on AMR.

Firstly, doctors prescribe antibiotics too often. For 97% of respondents, it has an impact, and for 53.3% of respondents, it has a definitely significant impact; for 95.6% of group A students, it has an impact, and for 55.7% of group A students it has a definitely significant impact; for 98.5% of group B students it has an impact, and for 50.4% it has a definitely significant impact (*p* = 0.650). 

Secondly, low awareness of the dangers resulting from the antimicrobial resistance (AMR). For 94.4% of respondents, it has an impact, and for 62.5% of respondents, it has a definitely significant impact; for 94.9% of group A students, it has an impact, and for 67.7% of group A students it has a definitely significant impact; for 94% of group B students it has an impact, and for 56.4% it has a definitely significant impact, (*p* = 0.023). 

Thirdly, the misuse of antibiotics in medicine. For 92.8% of respondents, it has an impact, and for 48.1% of respondents, it has a definitely significant impact; for 93% of group A students, it has an impact, and for 50% of group A students it has a definitely significant impact; for 92.5% of group B students it has an impact, and for 45.9% it has a definitely significant impact (*p* = 0.734). 

Other significant phenomena that impact the development of antibiotic resistance are the use of antibiotics in the production of farm animals, limited access to microbiological diagnostics, use of too low doses of the antibiotic, and low levels of hand hygiene. 

In the case of the use of antibiotics in livestock (husbandry), for 75.7% of subjects, it has an impact, and for 39.2% of subjects, it has a definitely significant impact; for 77.8% of group A students it has an impact, and for 36.7% of group A students it has a definitely significant impact; for 72.9% of group B students it has an impact, and for 42.1% it has a definitely significant impact (*p* = 0.928NS). 

In the case of limited access to microbiological diagnostics, for 74.9% of respondents, it has an impact, and for 24.7% it has a definitely significant impact; for 79.8% of group A students it has an impact, and for 29.1% of group A students it has a definitely significant impact; for 69.1% of group B students it has an impact, and for 19.5% it has a definitely significant impact (*p* = 0.010).

If too low a dose of the drug is used, for 72.5 of respondents it has an impact, and for 28.9% of respondents it has a definitely significant impact; for 69.7% of group A students it has an impact, and for 24.1% of group A students it has a definitely significant impact; for 76% of group B students it has an impact, and for 34.6% it has a definitely significant impact (*p* = 0.053NS).

In the case of a low level of hand hygiene, for 51.9% of respondents, it has an impact, and for 20.6% of subjects, it has a definitely significant impact; for 48.1% of group A students it has an impact, and for 15.8% of group A students it has a definitely significant impact; for 56.3% of group B students it has an impact, and for 26.3% it has a definitely significant impact (*p* = 0.069NS). 

The vast majority of the students surveyed (91.8%) agree that antibiotic resistance is a significant problem, with 93.7% of group A students and 89.5% of group B students agreeing. Less than eight percent (7.9%) stated it will become a problem in the future (6.3% vs. 9.8%). The majority of the students (77.6%) recognize that the problem of antibiotic resistance is global (74.5% vs. 81.2%). 

Half of the surveyed students (53.3%) have heard about the National Programme for Protection of Antibiotics conducted in Poland, while group B (74.4%) has heard about it much more often than group A (35.4%), *p* < 0.001. One-fifth of students have heard about the European Antibiotic Awareness Day campaign (26.1%), of which group A has heard about it 15.8%, and group B 38.3%, *p* < 0.001.

## 4. Discussion

Our study is the first one to evaluate the knowledge on antibiotics and antimicrobial resistance amongst Polish medical students. Our study has limitations. First, it is a single-institution audit, students represented only one medical university in Poland, so it has limited potential for impact outside the setting where it was performed. However, it is reasonable to assume that many universities would have similar results in several aspects after such an audit. Second, the response rate was 30% (289 out of 968), although some interesting data has been generated. This low response rate is consistent with other studies on similar topics [15,16,17].

Based on our results, it appears that Polish medical students participating in our study are aware of the consequences of antibiotic resistance. They recognize the main reasons for antibiotic resistance as follows: the overuse and misuse of antibiotics, lack of access to microbiological diagnostics, and low awareness of harmful consequences resulting from antibiotic resistance. Similar findings were reported in the UK in 2016 on students from 25 universities. Almost all participants indicated that the misuse of antibiotics promotes the spread of antibiotic-resistant bacteria [18]. Additionally, more than half of the students (63%) in a study conducted in France in 2012 considered that the overprescription of antibiotics, especially of those with a broad spectrum, contributes to antibiotic resistance development. The vast majority (96%) believed that antibiotic resistance could be a serious clinical problem in their future medical practice [16]. Concerns regarding the growing problem of antimicrobial resistance and the increasing threat to the effectiveness of infection treatment were also expressed by students of seven European medical universities (Dundee, Scotland; Geneva, Switzerland; Linkoping, Sweden; Ljublijana, Slovenia; Madrid, Spain, Nice, France; Oxford, England), who participated in the study conducted in 2012 [17]. A similar view was also expressed by dentistry students from all Polish medical universities in our previous study, and 54% of them considered that dentists overprescribe antibiotics [8].

The students participating in our study were divided into two groups, students of the years I–III and those of IV–VI. Such a division was chosen in order to evaluate whether, over the successive years of education, students increased their knowledge about the proper use of antibiotics and the problem of antibiotic resistance, and how their perception changes. It is particularly promising that more senior students (years IV–VI) less often indicated that it is appropriate to treat infections such as acute bronchitis with antibiotics. Senior students also were less likely (about 3%) to express the opinion that antibiotics are effective in treating influenza and colds, that is, typical viral infections. It is worrying that among graduating students, there are still those who believe that antibiotics affect viruses. A study on the knowledge of final-year dental students conducted in Poland in 2015 showed that about one-tenth of students declared the use of antibiotics for infections caused by viruses: influenza (7%) and cold (11%) [8]. Our earlier study on general population revealed that 36% of respondents believe that antibiotics are effective in the treatment of the cold, and as much as 49% against the influenza virus, this is much higher than for both dental and medical students [19].

The frequency and type of antibiotics prescribed depend primarily on physicians, dentists, and nurses in some countries. In the majority of countries, antibiotics are by law available by prescription only. Many studies have shown that obtaining antibiotics without a medical prescription is still a significant problem and contributes to improper and excessive use of this drug group [20,21]. Based on the above, we can distinguish two types of behaviors: purchasing an antibiotic without a prescription and taking the antibiotics remaining from previous therapies, family members, or friends. 

Among students participating in our study, almost half (46%) took an antibiotic in the last year. In the vast majority (89%), it was prescribed by a doctor; however, it should be noted that almost every tenth student was treated with the antibiotic remaining from the previous therapy or acquired in a pharmacy without a prescription. In a study conducted in 2016 among students of medicine, pharmacy, veterinary medicine, dentistry, physician associate and nursing. from 25 UK universities, more than one-third (86/242) took antibiotics during the last year, of which 3.5% received antibiotics from friends or family [18]. If we apply such behaviors to the general public, in a Eurobarometer survey conducted in 2018, one in three Europeans used antibiotics during the last year, of which 24% of Poland’s respondents declared such use [21]. 

The students participating in our study acknowledged the problem of antibiotic resistance at a global level (note that one in ten students had no opinion on this topic). In studies conducted in both Europe and the United States among medical students, it was noted that future doctors see the problem of antibiotic resistance at the national level rather than locally at the level of their hospital [16,17,22]. Similarly, the results presented in studies conducted among young physicians in Scotland, France, and the United States, who more often perceived antibiotic resistance as a problem at the national level, showed that they were less often considered essential in their everyday practice [23,24,25]. 

One of the essential elements of combating antibiotic resistance globally, recommended by international public health organizations and scientists, is to raise awareness of the proper use of antibiotics and the consequences of their misuse. This must apply to all sections of society, with particular reference to professionals authorized to prescribe antibiotics. A vast educational role is played by the “National Programme for Antibiotic Protection” (NPOA), implemented in Poland since 2004, which contains all the necessary elements in the area of medicine for the implementation of the so-called intersectoral co-ordinating mechanism (ICM), which, among other things, conducts educational activities aimed at both medical specialists and the general public. Since 2008, Poland has also participated in the information campaign European Antibiotic Awareness Day (EAAD) http://antybiotyki.edu.pl/edwa/ (4 February 2021). In our survey, we checked whether medical students have heard about these educational initiatives. More than half of the students participating in our study have heard about NPOA and one-fourth about EAAD. It points out that more older students than younger ones have heard about both activities.

An interesting position was taken by students of the last year of medicine participating in a study conducted in 2012 at seven medical universities in Europe. They mostly considered inappropriate or unnecessary prescribing of antibiotics as unethical professionally [17]. The ethical aspect of prescribing antibiotics by doctors is an extremely complex problem, which consists of such elements as socio-cultural conditions of the community in which the doctor works, competition between doctors, as well as conflict of interests between patients and doctors [26].

Knowledge about antibiotics, gained during the studies, impacts the attitudes of the studied students. In the Medical University of Warsaw study program, seminars and lectures on antibiotics are conducted within pharmacology classes. The basic issues are discussed, but without context of medical practice. As antibiotics are prescribed by doctors of every specialty, it seems that a particular emphasis should be placed on issues related to effective antibiotic therapy. A reasonable justification is the example of a study conducted among final-year students of medicine from 15 European countries, which showed higher accuracy of antibiotic prescription by students who participated in more training hours on prescribing antibiotics [27].

Many studies have confirmed the need for medical students to broaden their knowledge on the use of antibiotics, and individual studies have also pointed out the lack of certainty and understanding of medical students in the area of prescribing antibiotics [17,27,28]. The results of the research conducted among the students of the last year of medicine in 29 European countries indicated a varied need for further education in the use of antibiotics and the phenomenon of antibiotic resistance [29]. Students from Scandinavian countries (Sweden and Norway) felt the need for further education to a lower degree. In contrast, it was the highest among students from Portugal and Slovakia, which may indicate a lower priority placed on this subject in the curriculum of the latter. Students from Poland participating in the European survey and those in our study mostly expected further education in this area.

Students with a higher number of completed years of studies were significantly more likely to refuse a therapy prescribed by a doctor to them or to a family member. This is most likely due to their broader knowledge acquired during years of study. Another potential explanation for this behavior is whether the knowledge itself is sufficient and whether the denial of therapy is not due to a lack of trust in colleagues or the arrogant attitude of young doctors. The lack of ability to combine knowledge with everyday medical practice and the multifactorial nature of antibiotic therapy and resistance build-up is also important. As we know, in addition to the overuse and misuse of antibiotics contributing to the spread of resistant bacteria, it is important to keep sanitary conditions at a high standard, especially with regards to hand hygiene. In our study, almost half of the students (48%) indicated a low level of hand hygiene as not having a significant impact on the growth of antibiotic resistance. More than a quarter of French students reported hand hygiene as an insignificant cause of antibiotic resistance [16]. These responses indicate a lack of understanding of poor hand hygiene as a phenomenon contributing to the spread of bacterial pathogens, including resistant ones. 

Our study had an exploratory nature, and further studies are required in order to learn the attitudes of medical students from different medical universities around the world towards AMR.

## 5. Conclusions

Based on the distribution of the received responses, our study demonstrated that there is a need to increase the number of hours of antibiotic therapy in medical schools and the students themselves are drawing attention to this need.

Future physicians should be able to graduate from medical school with the knowledge that allows them to make the right decisions regarding all aspects of antibiotic therapy, and this topic should be given more priority in medical studies, across EU.

We suggest focusing not only on the curriculum but also on the attitudes of academic teachers responsible for passing on knowledge to future generations of doctors, which can significantly impact the trust in the medical community. Knowledge should go hand in hand with an adherence to recommendations in clinical work and actions to prevent the rise of antibiotic resistance. 

## Figures and Tables

**Table 1:** Knowledge and attitude towards antibiotic therapy.

	Year of Study 1–3 n (%)	Year of Study 4–6 n (%)	Total n (%)	*p*-Value sig. *	Adjusted ORs (95% Cl)
	*n* = 158	*n* = 133	*n* = 291		
**How did you obtain the last course of antibiotics that you used?**					
From a medical prescription—family practitioner	89 (56.6)	57 (43.2)	146 (50.3)	**0.022**	**0.580 (0.364–0.925)**
From a medical prescription—physician of other specialty	46 (29.3)	44 (33.3)	90 (31.1)	0.466	1.209 (0.734–1.991)
From a medical prescription—dentist	10 (6.4)	11 (8.3)	21 (7.7)	0.524	1.331 (0.547–3.239)
Without prescription from a pharmacy	2 (1.3)	2 (1.5)	4 (1.4)	0.862	1.149 (0.158–8.387)
Leftovers from a previous therapy	6 (3.8)	5 (3.8)	11 (3.8)	0.986	1.015 (0.300–3.440)
From friends/family member	3 (1.3)	10 (7.6)	13 (4.2)	**0.008**	**6.315 (1.354–29.461)**
Other	2 (1.3)	3 (2.3)	5 (1.7)	0.419	1.816 (0.299–11.045)
What was the reason for your last intake of antibiotics? **					
Pharyngitis	42 (26.8)	30 (22.7)	72 (24.9)	0.431	0.802 (0.467–1.377)
Acute bronchitis	22 (14.0)	15 (11.4)	37 (12.8)	0.502	0.789 (0.391–1.594)
Sore throat	27 (17.2)	8 (6.1)	35 (12.1)	**0.004**	**0.310 (0.136–0.709)**
Sinusitis	9 (5.7)	15 (11.4)	24 (8.3)	0.084	2.135 (0.899–5.070)
Cough	18 (8.9)	6 (7.0)	24 (8.3)	0.594	1.212 (0.525–2.796)
Otitis	14 (8.9)	10 (7.6)	24 (8.3)	0.681	0.837 (0.359–1.953)
Pneumonia	13 (8.3)	9 (6.8)	22 (7.6)	0.641	0.811 (0.335–1.963)
Urinary tract infection	8 (5.1)	14 (10.6)	22 (7.6)	0.078	2.222 (0.901–5.479)
Common cold	11 (7)	8 (6.1)	19 (6.6)	0.747	0.862 (0.335–2.216)
Flu	7 (4.5)	1 (0.8)	8 (2.8)	0.056	0.163 (0.020–1.352)
Diarrhea	0 (0)	4 (3.0)	4 (1.4)	**0.028**	-
**When antibiotics s** hould be prescribed as first-line treatment? **					
Pneumonia	137 (86.7)	118 (88.7)	255 (87.6)	0.603	1.198 (0.590–2.433)
Borreliosis	112 (70.9)	114 (85.7)	226 (77.7)	**0.002**	2.463 (1.355–4.475)
Urinary tract infection	109 (69.0)	110 (82.7)	219 (75.3)	**0.010**	2.252 (1.269–3.999)
Otitis	102 (64.6)	77 (57.9)	179 (61.5)	0.306	0.742 (0.461–1.194)
Acute bronchitis	105 (66.5)	26 (19.5)	131 (45.0)	<**0.001**	0.123 (0.071–0.211)
Sinusitis	64 (40.5)	44 (33.1)	108 (37.1)	0.192	0.723 (0.447–1.171)
Sore throat	39 (24.7)	21 (15.8)	60 (20.6)	0.062	0.573 (0.318–1.033)
Toothache	4 (2.5)	4 (3.0)	8 (2.7)	0.805	1.199 (0.294–4.891)
Diarrhea	4 (2.5)	3(2.3)	7 (2.4)	0.878	0.889 (0.195–4.048)
Flu	5 (3.2)	2 (1.5)	7 (2.4)	0.357	0.476 (0.090–2.510)
Pharyngitis	4 (2.5)	1 (0.8 )	5 (1.7)	0.283	0.283 (0.031–2.581)

* sig.—statistically significant (in bold); ** the table contains selected answers, showing the difference between year of the study, 1–3 and 4–6.

**Table 2:** Impact of education on attitudes towards antibiotics.

	Year of Study 1–3 n (%)	Year of Study 4–6 n (%)	Total n (%)	*p*-Value sig. *
	*n* = 158	*n* = 133	*n* = 291	
**Were you taught during your studies about the growing problem of microbial resistance?**				
Yes	137 (6.7)	132 (99.2)	269 (92.4)	**<0.001**
No	21 (13.3)	1 (0.8)	22 (7.6)	
**Did the knowledge you gained about antibiotics during your medical studies have an impact on negating the antibiotic therapy ordered by a doctor in case of your disease?**				
Yes	26 (16.5)	43 (32.3)	69 (23.7)	**0.002**
No	132 (83.5)	90 (67.7)	222 (76.3)	
**Did the knowledge you gained about antibiotics during your medical studies have an impact on negating the antibiotic therapy ordered by a doctor when someone in your family got sick?**				
Yes	48 (30.4)	71 (53.4)	119 (40.9)	**<0.001**
No	110 (69.6)	62 (46.6)	172 (59.1)	

* sig.—statistically significant (in bold).

## Data Availability

The data presented in this study are available on request from the corresponding author.

## Supplementary Materials

The following are available online at https://www.mdpi.com/article/10.3390/ijerph18083930/s1, File S1: Full original questionnaire in English.
