# Supplementary material for: Knowledge-Based Attitudes of Medical Students in Antibiotic Therapy and Antibiotic Resistance. A Cross-Sectional Study
Source: Int J Environ Res Public Health. 2021 Apr 8;18(8):3930. doi: 10.3390/ijerph18083930 (PMC8068920; doi:10.3390/ijerph18083930)
Supplement: Supplementary file 1 [file ijerph-18-03930-s001.pdf]

ATTITUDES OF MEDICAL UNIVERSITY OF WARSAW STUDENTS TOWARD ANTIBIOTICS

Dear Sir/Madam,
The following questionnaire is a key stage of a sociological research process conducted jointly by the Medical University of Warsaw, the National Medicines Institute and the University of Warsaw, and its aim is to find out the attitudes of WUM students towards antibiotics.
Thank you for taking part in this survey.
Each of your answers is very valuable.
Unless otherwise stated at the question, please mark <u>only one answer</u> of your choice. <u>The survey is anonymous</u>.

<u>**METRIC DATA**</u>

A. Gender

☐ female ☐ male

B. Year of study

☐ 1st year ☐ 2nd year ☐ 3rd year ☐ 4th year ☐ 5th year ☐ 6th year

<u>**QUESTIONS**</u>

1. When was the last course of an atibiotics?

a. ☐ in the last month
b. ☐ 1-2 months ago
c. ☐ 3-6 months ago
d. ☐ 7-12 months ago
e. ☐ more than 1 year ago but not more than 2 years ago
f. ☐ more than 2 years ago but not more than 5 years ago
g. ☐ more than 5 years ago
h. ☐ never (*please go to the question 6*)

2. How did you obtain the last course of antibiotics that you used?

a. ☐ from a medical prescription – family practitioner
b. ☐ from a medical prescription – physician of other specialty *(which one?)* ………………………
c. ☐ from a medical prescription – dentist
d. ☐ form a medical prescription - nurse
e. ☐ without prescription form a pharmacy
f. ☐ leftovers form a previous pharmacy
g. ☐ form friends/family member
h. ☐ other *(which one?)* ……………………………………………………

3. What was the reason for your last intake of antibiotics (*more than one answer may be marked*)

a. ☐ common cold
b. ☐ cough
c. ☐ sore throat
d. ☐ toothache
e. ☐ flu
f. ☐ pharyngitis
g. ☐ acute bronchitis
h. ☐ pneumonia
i. ☐ urinary tract infection
j. ☐ diarrhea
k. ☐ otitis
l. ☐ borreliosis
m. ☐ sinusitis
n. ☐ shielding before surgery
o. ☐ other *(wich one?)* ………………………………………………………………………
p. ☐ I do not remember

4. **Have you used the whole antibiotic?**
   a. ☐ yes *(please go to the question no. 6)*
   b. ☐ no

5. **For what reason you have not used the whole antibiotic?**
   a. ☐ I am still in therapy
   b. ☐ my doctor/dentist has advised me to stop treatment
   c. ☐ I felt better
   d. ☐ due to side effects (e.g. diarrhoea, rash)
   e. ☐ the antibiotic was not effective
   f. ☐ I forgot
   g. ☐ I wanted to save some for later
   h. ☐ I felt like drinking alcohol
   i. ☐ due to other medical reasons *(which one?)* ....
   j. ☐ due to other non-medical reasons *(which one?)* ....

6. **How would you rate your knowledge about antibiotics?** (*please indicate your answer on a scale where 1 means "very bad" and 6 means "very good")*

   ☐1 ☐2 ☐3 ☐4 ☐5 ☐6

7. **How would you rate the knowledge of other WUM students about antibiotics?** (*please indicate your answer on the scale where 1 means "very bad" and 6 means "very good"*)

   ☐1 ☐2 ☐3 ☐4 ☐5 ☐6

8. **How would you rate the knowledge of Poles about antibiotics?** (*please indicate your answer on the scale where 1 means "very bad" and 6 means "very good"*)

   ☐1 ☐2 ☐3 ☐4 ☐5 ☐6

9. **In your opinion, antibiotics should be prescribed as first-line treatment in the case of** (*more than one answer may be marked*)
   a. ☐ common cold
   b. ☐ cough
   c. ☐ sore throat
   d. ☐ toothache
   e. ☐ flu
   f. ☐ pharyngitis
   g. ☐ acute bronchitis
   h. ☐ pneumonia
   i. ☐ urinary tract infection
   j. ☐ diarrhea
   k. ☐ otitis
   l. ☐ borreliosis
   m. ☐ sinusitis
   n. ☐ other *(which one?)* ....
   o. ☐ I do not know

10. **Were you taught during your studies about the increasing problem of microbial resistance?**
   a. ☐ yes
   b. ☐ no

11. **Did your medical studies make you more knowledgeable about the use of antibiotics?** (*please mark your answer on the scale where 1 means "definitely not" and 6 means "definitely yes"*)

☐1 ☐2 ☐3 ☐4 ☐5 ☐6

12. **Did the knowledge about antibiotics you gained during medical studies have had an impact on negating the antibiotic therapy ordered by a doctor in case of:**

13a. your disease

a. ☐ yes
b. ☐ no

13b. disease of someone of your family

a. ☐ yes
b. ☐ no

13. **Below are some views on antibiotics. Please indicate on the scale how much you agree or disagree with each of them** (*where 1 means "strongly disagree" and 6 means "strongly agree"*)

a. antibiotics are effective against viruses

☐1 ☐2 ☐3 ☐4 ☐5 ☐6

b. antibiotics are effective against bacteria

☐1 ☐2 ☐3 ☐4 ☐5 ☐6

c. inappropriate use of antibiotics can cause microorganisms to become resistant to them

☐1 ☐2 ☐3 ☐4 ☐5 ☐6

d. the use of antibiotics will make people resistant to them

☐1 ☐2 ☐3 ☐4 ☐5 ☐6

e. the use of antibiotics often causes side effects (e.g. diarrhoea, headaches, stomach pains, allergies)

☐1 ☐2 ☐3 ☐4 ☐5 ☐6

f. antibiotics are effective against the common cold

☐1 ☐2 ☐3 ☐4 ☐5 ☐6

g. antibiotics are effective for influenza

☐1 ☐2 ☐3 ☐4 ☐5 ☐6

h. doctors often prescribe antibiotics unnecessarily

☐1 ☐2 ☐3 ☐4 ☐5 ☐6

i. bacteria communicate antibiotic resistance to each other

☐1 ☐2 ☐3 ☐4 ☐5 ☐6

14. In which of the following sources of information, and to what extent, would you seek information on the use of an antibiotic, if it were you or someone close to you? (*please indicate your answer on the scale, where 1 means "not at all" and 6 means "to a considerable extent"*)

14a. at the doctor

☐1 ☐2 ☐3 ☐4 ☐5 ☐6

14b. on the Internet

☐1 ☐2 ☐3 ☐4 ☐5 ☐6

14c. in the literature (in hard copy outside the Internet)

☐1 ☐2 ☐3 ☐4 ☐5 ☐6

14d. in the drug's leaflet

☐1 ☐2 ☐3 ☐4 ☐5 ☐6

15. Would you like to increase your knowledge regarding the use of antibiotics?

a. ☐ definitely yes
b. ☐ rather yes
c. ☐ rather no *(please go to the question no. 17)*
d. ☐ zdecydowanie nie *(please go to the question no. 17)*

16. If yes, which of the following topics would you like to explore: (*more than one answer may be marked*)

a. ☐ general principles for the rational use of antibiotics
b. ☐ information on antibiotic resistance of microorganisms causing respiratory tract infections
c. ☐ information on antibiotic resistance of microorganisms causing urinary tract infections
d. ☐ information on antibiotic resistance of microorganisms causing skin and subcutaneous tissue infections
e. ☐ information on antibiotic resistance of microorganisms causing infections of the digestive system
f. ☐ information on the micro-organisms that cause oral infections
g. ☐ information on the possibilities for microbiological diagnosis of infections
h. ☐ information on the reasons for failure of antibiotic therapy
i. ☐ other *(which one?)* ……………………………………………………

17. If your doctor advised you to take an antibiotic, then:

a. ☐ I would start the treatment with full confidence in the doctor
b. ☐ I would check the information on the antibiotic and the recommendations in some other source before starting the treatment
c. ☐ I would mainly trust myself and my knowledge about the drug

18. In your opinion, the antibiogram should be ordered before each antibiotic administration? *(please indicate your answer on the scale where 1 means "strongly disagree" and 6 means "strongly agree")*

☐1 ☐2 ☐3 ☐4 ☐5 ☐6

19. In your opinion, is the issue of antibiotic resistance of microorganisms a major problem?

a. ☐ yes
b. ☐ not at the present, but will become a problem in the future *(please go to the question no. 21)*
c. ☐ no *(please go to the question no. 21)*

20. If yes, please indicate at what level do you perceive the problem of antibiotic resistance?

a. ☐ at regional level
b. ☐ at country level
c. ☐ at european level
d. ☐ at global level
e. ☐ I do not know, it's hard to say

21. Have you heard of the National Programme for the Protection of Antibiotics in Poland?

a. ☐ yes
b. ☐ no

22. Have you heard of the European Antibiotic Awareness Day?

a. ☐ yes
b. ☐ no

23. To what extent do you think that the attitudes/behaviors/indications listed below have an impact on the development of antibiotic resistance? *(please mark your answer on the scale where 1 means "little influence" and 6 means "considerable influence")*

a. excessive use/prescription of antibiotics by doctors

☐1 ☐2 ☐3 ☐4 ☐5 ☐6

b. excessive use/prescription of antibiotics by dentists

☐1 ☐2 ☐3 ☐4 ☐5 ☐6

c. excessive use/prescription of antibiotics by nurses

☐1 ☐2 ☐3 ☐4 ☐5 ☐6

d. low awareness of the dangers of the antibiotic resistance phenomenon

☐1 ☐2 ☐3 ☐4 ☐5 ☐6

e. too long an antibiotic therapy

☐1 ☐2 ☐3 ☐4 ☐5 ☐6

f. use of too low doses of antibiotic

☐1 ☐2 ☐3 ☐4 ☐5 ☐6

g. use of antibiotics for fattening livestock

☐1 ☐2 ☐3 ☐4 ☐5 ☐6

h. limited access to microbiological diagnostics

☐1 ☐2 ☐3 ☐4 ☐5 ☐6

i. misuse of antibiotics in veterinary medicine

☐1 ☐2 ☐3 ☐4 ☐5 ☐6

j. low level of hand hygiene

☐1 ☐2 ☐3 ☐4 ☐5 ☐6

k. overuse of antibiotics in medicine

☐1 ☐2 ☐3 ☐4 ☐5 ☐6

24. In your opinion, are there currently standards in medicine and dentistry for the use of antibiotics in specific clinical situations? *(please mark your answer on the scale where 1 means "definitely not" and 6 means "definitely yes")*

☐1 ☐2 ☐3 ☐4 ☐5 ☐6
